# Editorial: Occupation and cancer: new insights into burden, risk factors, and prevention

**DOI:** 10.3389/fpubh.2023.1343952

**Published:** 2024-01-04

**Authors:** Balázs Ádám, Alberto Modenese, Tom Loney

**Affiliations:** ^1^Institute of Public Health, College of Medicine and Health Sciences, United Arab Emirates University, Al Ain, United Arab Emirates; ^2^Department of Biomedical, Metabolic and Neural Sciences, University of Modena and Reggio Emilia, Modena, Italy; ^3^College of Medicine, Mohammed Bin Rashid University of Medicine and Health Sciences, Dubai Health, Dubai, United Arab Emirates

**Keywords:** occupational cancer, carcinogenic exposure, prevention, lung cancer, mesothelioma, non-melanoma skin cancers, bladder cancer, breast cancer

Occupational cancers (OC) are the result of exposure to carcinogenic agents at the workplace. The most frequent types of OC are lung cancer, mesothelioma, bladder cancer, and non-melanoma skin cancers (NMSC) (1, 2). More than 40 years ago, Doll and Peto (3) estimated, with a relatively large degree of uncertainty, that ~4% of all cancer cases were attributable to occupational exposures. Although job characteristics and occupational exposures have changed considerably during the past four decades, this estimation is still widely accepted with the caveat that updated estimates are urgently needed. Nevertheless, only a fraction of OCs are recognized and recorded in most countries. With the possible exception of mesothelioma, all the other OCs are largely under-reported as occupational diseases to the national health authorities. Indeed, many countries, especially developing countries, where exposures to occupational carcinogens may be higher, have not yet established cancer registries that collect data on occupational history. An additional challenge of discovering occupational etiology relates to the fact that cancer is a disease with a long preclinical phase and many OCs are diagnosed after retirement. Hence, this Research Topic aimed to deepen and widen knowledge on OC, in order to raise awareness among all interested stakeholders, including workers, occupational health and safety (OHS) professionals, and policymakers, with the overall goal of preventing these occupational diseases.

Considering more “traditional” OCs, such as mesothelioma and lung cancers, Grignani et al. highlight the challenges of identifying possible biomarkers of early alterations associated with mesothelioma in asbestos-exposed workers. Specifically, Grignani et al. evaluated single nucleotide polymorphisms as possible markers of susceptibility, with no promising results on their utilization. Regarding lung cancer, Marsh and Kruchten analyzed data from the US National Cancer Institute to explore the relationship between acrylonitrile exposure, hypothesized to alter iron metabolism, and mortality in lung and bladder cancers. Their analysis did not provide additional evidence to support the association. Finally, van der Linden et al. estimate the 10-year risk of lung and breast cancer by occupation in Switzerland. Interestingly, the authors reported that men involved in jobs they categorized as “elementary professions” (e.g., agriculture, industry, and crafts) had a higher 10-year risk of lung cancer compared to other workers, whilst women working in intermediate professions (e.g., administrative employees) had the highest risk for lung cancer. However, the highest risk for developing breast cancer was amongst women in managerial professions. Of note, smoking tobacco created a greater change in 10-year cancer risk than occupation for both sexes. This finding reinforces the need for workplace wellness programs supporting healthy lifestyle behaviors in addition to annual occupational health assessments for employees exposed to workplace carcinogens and other health hazards.

There is a growing body of research linking specific occupations to the development of breast cancer. Canu et al. further this work by adapting the life course exposome model to explore the role of the “worksome” (viz. physical and psychosocial exposures and effects derived from work and working conditions) on breast cancer survival. The study utilized cancer registry records to match women diagnosed with primary invasive breast cancer between 1990 and 2014 with the Swiss National Cohort. Of note, women in elementary occupations with low skill levels had the lowest survival rates and professionals with the highest skill level, generally in senior management positions or independent professions, had the highest survival rates. These findings suggest a possible protective effect of certain “worksomes” that warrant further investigation in larger and more diverse population-based cohorts with data on a wider range of covariates to account, e.g., for access to better health services and better compliance.

Of course, there are certain occupations that are inherently risky and potentially expose the worker to a wide range of physical and chemical hazards that could lead to cancer. Firefighting is one such occupation. Kunz et al. conducted a cross-sectional study among female firefighters in 12 countries and found that those with cancer had been in the fire service longer and had more career fires and toxic exposures. Again, firefighters with cancer reported more tobacco use and this highlights the need for further research to explore the probably synergistic effect of occupational carcinogenic exposures and tobacco use on the development of different cancers. In view of the limitations of cross-sectional designs (e.g., temporal bias), future studies employing prospective cohort designs are required to quantify the risk of specific workplace exposures associated with certain occupational diseases.

The final two papers in this Research Topic focus on an important but currently overlooked and under-reported work-related neoplasm, namely, skin cancer caused by occupational exposure to solar ultraviolet radiation (UVR). Symanzik and John provide some insightful perspectives on the current challenges of quantifying cumulative solar UVR exposure to accurately estimate the risk of occupational skin cancer. This article dovetails with a novel approach proposed by Paulo et al. to utilize personal electronic dosimeters integrated with a digital platform to assess occupational solar UVR doses of outdoor workers in Portugal. Moreover, the study plans to develop a digital platform for the workers that will use their personal solar UVR exposure data and communicate their risk of developing skin cancer based on their skin type.

We hope that you find the seven articles contained in this Research Topic on Occupation and Cancer intellectually stimulating and that you have become more aware of the relationship between specific occupational exposures and certain cancers, and possibly, you feel motivated to embark on research in the area of the “worksome”.

## Author contributions

BÁ: Conceptualization, Writing—original draft, Writing—review & editing. AM: Conceptualization, Writing—original draft, Writing—review & editing. TL: Conceptualization, Writing—original draft, Writing—review & editing.
